# Design of a biomimetic, small-scale artificial leaf surface for the study of environmental interactions

**DOI:** 10.3762/bjnano.13.83

**Published:** 2022-09-13

**Authors:** Miriam Anna Huth, Axel Huth, Lukas Schreiber, Kerstin Koch

**Affiliations:** 1 Faculty of Life Sciences, Rhine-Waal University of Applied Sciences, Marie-Curie-Str. 1, 47533 Kleve, Germany; 2 IZMB, Department of Ecophysiology, University of Bonn, Kirschallee 1, 53115 Bonn, Germany

**Keywords:** recrystallization, surface properties, wax composition, wetting, wheat

## Abstract

The cuticle with its superimposed epicuticular waxes represents the barrier of all aboveground parts of higher plant primary tissues. Epicuticular waxes have multiple effects on the interaction of plants with their living and non-living environment, whereby their shape, dimension, arrangement, and chemical composition play significant roles. Here, the ability of self-assembly of wax after isolation from the leaves was used to develop a small-scale wax-coated artificial leaf surface with the chemical composition and wettability of wheat (*Triticum aestivum*) leaves. By thermal evaporation of extracted plant waxes and adjustment of the evaporated wax amounts, the wettability and chemical character of the microstructure of the surface of wheat leaves were transferred onto a technical surface. For the use of these artificial leaves as a test system for biotic (e.g., germination of fungal pathogens) and non-biotic (e.g., applied surfactants) interactions on natural leaf surfaces, the chemical composition and the wetting behavior should be the same in both. Therefore, the morphology, chemistry, and wetting properties of natural and artificial surfaces with recrystallized wax structures were analyzed by scanning electron microscopy, gas chromatography–mass spectrometry, and by the determination of water contact angles, contact angle hysteresis, and tilting angles. Wheat leaves of different ages were covered exclusively with wax platelets. The extracted wheat wax was composed of alcohols, aldehydes, esters, and acids. The main component was 1-octacosanol. The waxes recrystallized as three-dimensional structures on the artificial surfaces. The three tested wetting parameters resembled the ones of the natural surface, providing an artificial surface with the chemical information of epicuticular waxes and the wetting properties of a natural leaf surface.

## Introduction

### Cuticle

**Figure 1:**
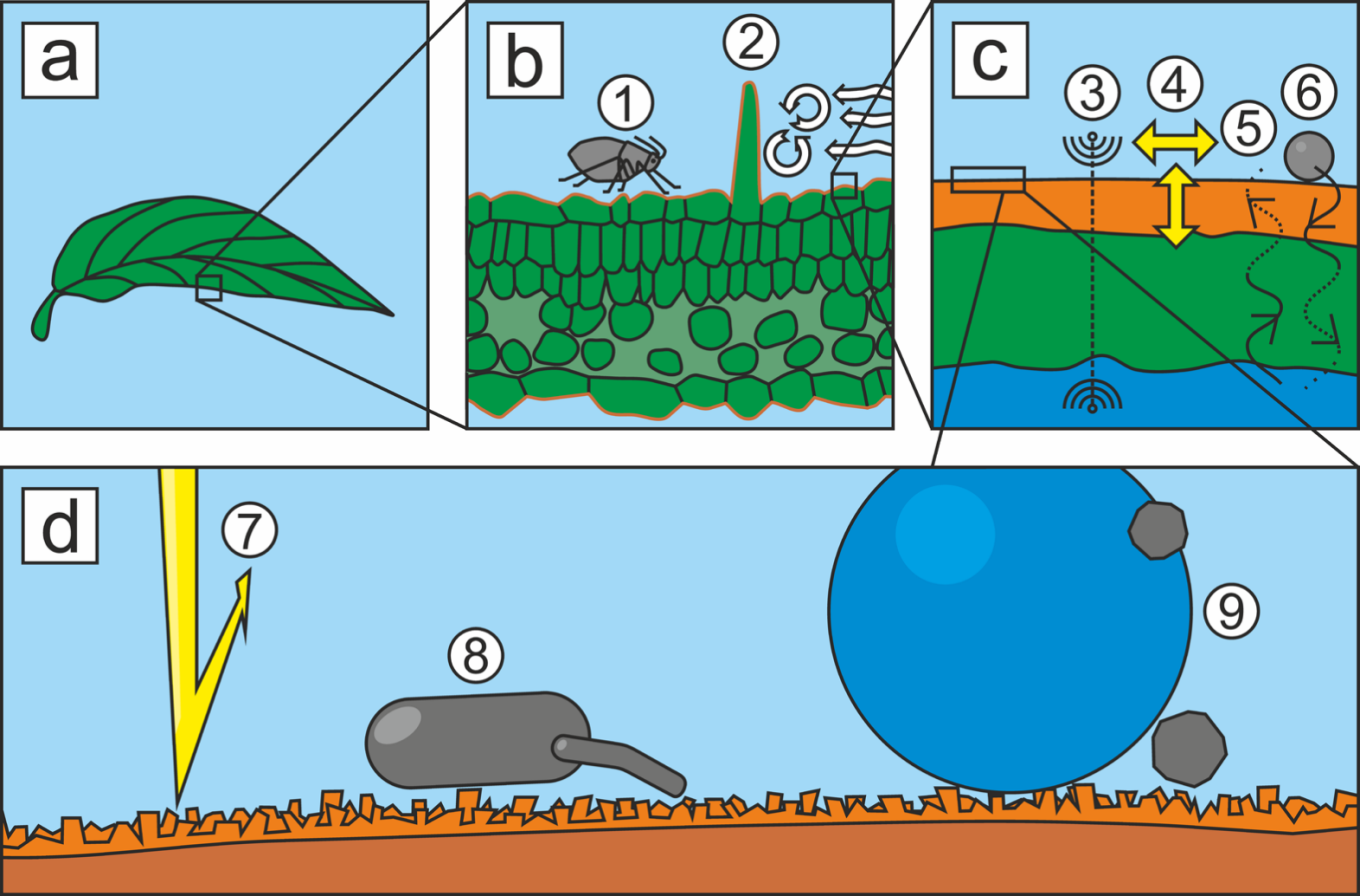
Drawings of a leaf surface at different magnifications and main functions of the cuticle. (a) Leaf, (b–d) leaf cross sections in increasing magnifications; (c) orange: cuticle, green: cell wall, blue: cytoplasm, (d) orange: epicuticular wax crystals, brown: cuticle. (1) Reduction of arthropod pest attachment, (2) complex functions of trichomes, for example, generation of air turbulences, (3) host-pathogen recognition/signaling for cell development, (4) resistance to mechanical stress, (5) control of water loss, (6) control of solute uptake, (7) protection from hazardous radiation, (8) reduction of spore, bacterial, and viral attachment, (9) self-cleaning ability (modified after [9]).

One of the largest interfaces on earth is formed by thin layers that are a few nanometers to micrometers thin, namely the wax layers of the plant cuticle [1]. The plant cuticle is a thin extracellular membrane superimposed on the epidermal cells of all higher, non-woody, aboveground plant surfaces. It is basically composed of an insoluble polymeric matrix, cutin, and soluble hydrophobic waxes. The plant cuticle is known to have a variety of vital functions (Figure 1). Among other things, it protects against herbivores and pathogens, provides mechanical stability, reflects harmful UV radiation [2–6], and mainly protects the plant from desiccation [7–8]. The cuticular waxes contribute significantly to this barrier function.

### Plant waxes

Plant waxes are complex mixtures of long-chain aliphatic and cyclic hydrocarbon compounds with different functional groups [10]. Typical wax components are fatty acids, alcohols, ketones, aldehydes, and triterpenes, but the exact wax composition is species- and organism-specific [11]. The waxes are differentiated into intracuticular waxes, which are incorporated into the cutin matrix, and epicuticular waxes, which are deposited on the polymer. Intra- and epicuticular waxes differ not only in their localization, but also in their chemical composition [12–13]. Epicuticular waxes form the outermost layer of the plant cuticle and, thus, the first contact site of the plant with its biotic and abiotic environment. They form a thin crystalline wax film (less than 1 µm) and often various three-dimensional emerging wax crystals in the nano- and micrometer range (0.5–100 µm) [14–16]. Recrystallization of extracted plant waxes has shown that the diverse microstructures of epicuticular waxes arise by self-assembly and that the micromorphology of wax structures is largely determined by their chemical composition [15,17–23]. Platelets, which mostly have a high primary alcohol content, are among the most common wax structures. An overview of the morphology of various wax structures has been given by Barthlott and co-workers [24].

### Wetting

The wettability of leaves plays an important role in the interaction of plants with the environment, such as the interaction with pathogens and the formation of biofilms [25–26]. A measure of the degree of wetting is the contact angle θ (CA). It describes the angle between the liquid–vapor interface and the liquid–solid interface. According to the CA, surfaces can be classified as superhydrophilic (0° < θ < 10°), hydrophilic (10° ≤ θ < 90°), hydrophobic (90° ≤ θ < 150°), and superhydrophobic (150° ≤ θ < 180°) [27]. Due to its chemical composition, the cuticle is generally a hydrophobic surface. However, plant surfaces show a large variability of cell shapes, surface structures, and hierarchical structures (a combination of micro- and nanostructures) [27]. Due to this structural diversity and different chemical modifications, plant surfaces can have different wetting properties, ranging from superhydrophobic to superhydrophilic [28]. An overview of the diverse microstructures and their influence on the wettability of plant surfaces is given by several reviews [27,29–31].

#### Wetting states

Young was the first to formulate a description of the wetting state in 1805. According to Young's equation, the CA depends on the three interfacial tensions liquid–solid, liquid–gas, and solid–gas [32]. He describes an ideal static CA on a homogeneous, smooth, non-deformable, and inert surface. Natural and technical surfaces hardly correspond to these ideal conditions. Wenzel [33] and Cassie and Baxter [34] studied CAs on rough surfaces, assuming that the liquid penetrates perfectly into the depressions of the surface (homogeneous wetting). Cassie and Baxter [34] describe heterogeneous wetting in which the liquid does not penetrate the depressions of the surface. Air pockets form under the liquid, which reduce the contact between the solid surface and the liquid. In nature, ideal Wenzel or Cassie–Baxter wetting stages rarely occur. However, often so-called mixed wetting stages exist [35].

#### Contact angle hysteresis and tilting angle

The wetting stages are closely related to the contact angle hysteresis (CAH) and the tilting angle (TA). Both quantities are therefore used to adequately describe the wettability of rough and heterogeneous surfaces. CAH is a qualitative measure of the mobility of a droplet on a surface [36]. The larger the hysteresis, the harder a droplet rolls off [37]. CAH is defined as the difference between the advancing and the receding CA. It can be determined for a droplet with varying volume or for a droplet on an inclined plane. The angle of inclination at which a droplet starts to roll off is called the TA α. Superhydrophobic surfaces with a low TA (α < 10°) and a small CAH have the ability to self-clean [9]. This property is also known as the Lotus effect, named after the best-known example of a self-cleaning surface, the leaf of the lotus plant (*Nelumbo nucifera* Gaertn., Nelumbonaceae).

### Biomimetic surfaces

The wettability properties of plant surfaces have often been a source of inspiration for the development of biomimetic materials. For example, biomimetic surfaces offer the possibility to study interfacial phenomena of plant surfaces such as the splash behavior of liquids or the adhesion of insects under laboratory conditions [38–39]. In the past, long-chain hydrocarbons as well as native wax extract were recrytallized to mimic the native leaf structures and their associated properties [21,39–42]. Different methods of recrystallization have been developed and used in previous studies [17,20–21,43–46]. A fast method, for example, is recrystallization from a solution. However, this process has the disadvantage that it can lead to a heterogeneous distribution of the wax mass and to a variety of structures that often do not correspond to the original wax type [20,42]. Due to the ring-shaped accumulation and the resulting pattern, this effect is called the “coffee drop effect” [47–48]. This undesirable effect can be avoided by the solvent-free process of physical vapor deposition (PVD), so that a homogeneous distribution of the wax masses can be achieved [42].

### Aim of this study

In the present study, the wettability and chemical character of a natural leaf surface were transferred to technical surfaces by the process of PVD. Wheat, one of the most important crops worldwide, served as the biological model. The artificial surfaces imitating the leaves should have the same chemical and wetting properties as leaf surfaces. To prove these criteria, the chemical composition of the native and transferred wax extracts, the morphology of native and recrystallized wax crystals and the wetting properties of native and artificial surfaces were analyzed. Such artificial surfaces allow for the study of biotic interactions, for example, pathogen interactions, but also non-biotic interactions such as the behavior of liquids and surfactants used in agricultural spraying applications. An artificial test system is independent of the environmental influences to which plants in a field are exposed. It can be developed in large numbers with little resources towards the detailed understanding of interfacial effects under laboratory conditions.

## Experimental

### Plant material

Wheat plants (*Triticum aestivum* L., Poaceae, variety Ponticus, Strube D&S GmbH, Söllingen, Germany) were cultivated in the greenhouse (GH plants, temp. day: 18 °C, temp. night: 12 °C) in pots with arable soil, sand, and universal soil (1:1:2). The plants were irrigated and fertilized (2.5% Universol Blue 18-11-18 + 2.5 MgO + TE, Everris International BV, Heerlen, Netherlands) manually once a week. In addition, outdoor plants (OD plants) were cultivated in the experimental garden of the Rhine-Waal University of Applied Sciences, Kleve, Germany, under preferably realistic cultivation conditions in order to be able to compare their wax morphology and chemistry with those of the plants cultivated under laboratory conditions. The plants were sown by hand. The row spacing was approx. 35 cm and the grain spacing approx. 10 cm. Fertilizer was applied once with saltpeter granules (YaraLiva CALCINIT, Yara GmbH & Co. KG, Dülmen, Germany). A total of about 100 plants each were grown in the greenhouse and outdoors.

In all conducted experiments, the second, third, and fourth leaves were harvested when the fifth leaf was fully developed (Supporting Information File 1, Figure S1). The leaves at the second position were the oldest and the leaves at the fourth position were the youngest ones. Chemical composition, wax structures, and wettability were characterized separately for leaves of different ages in order to find or exclude possible variations (see following sections). In the following, the leaves are referred to as leaf 2, 3, and 4 according to their positions on the plant.

### Development of artificial leaf surfaces

**Figure 2:**
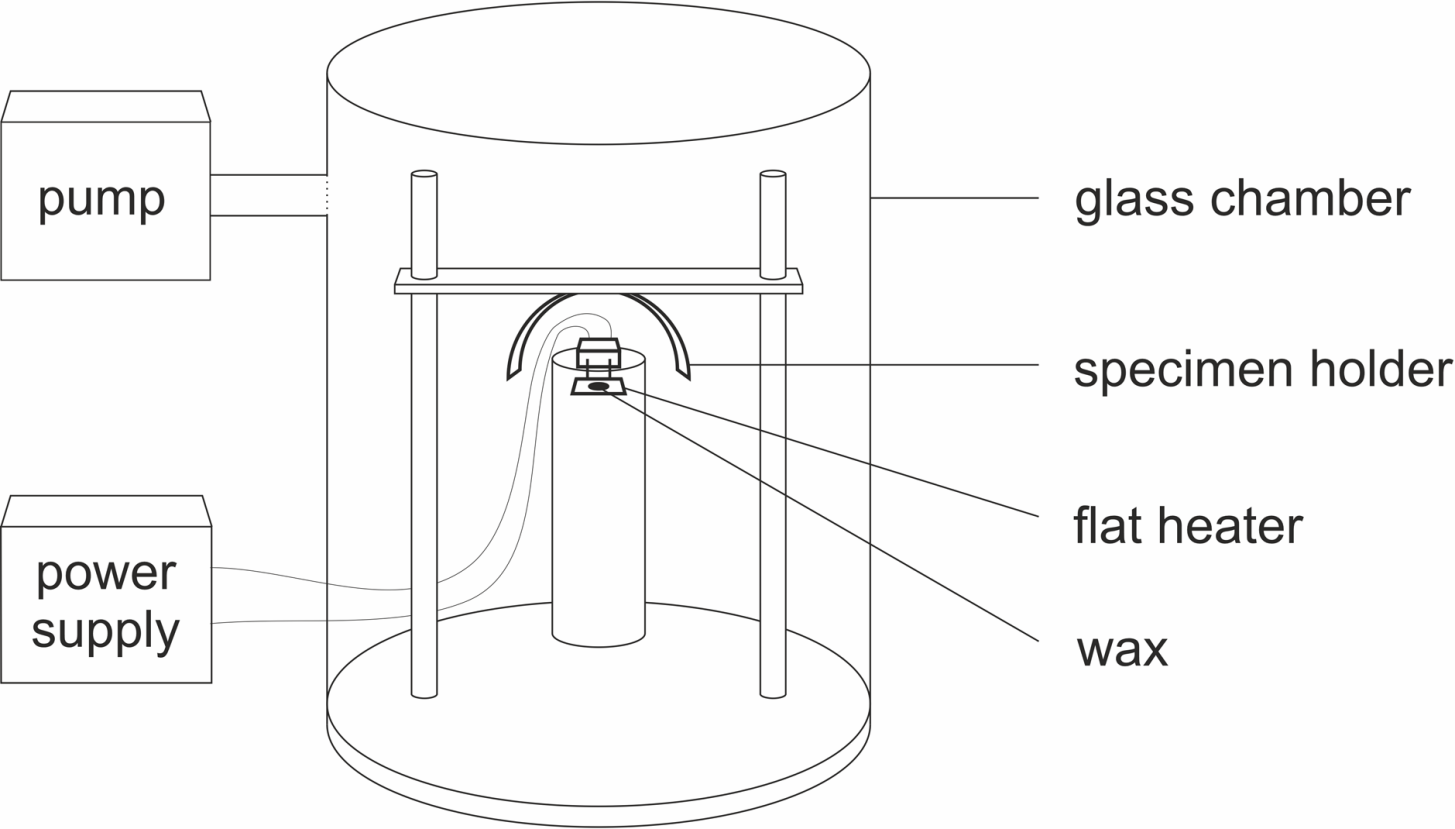
Schematic drawing of the device for wax vaporization by PVD.

Wax for recrystallization was extracted from wheat leaves of outdoor plants by immersing the leaves in chloroform (99.9% HPLC grade, Carl Roth GmbH und Co. KG, Karlsruhe, Germany) for 20 s. The extracts were then filtered (ROTILABO type 113A, 110 mm diameter, Carl Roth GmbH und Co. KG, Karlsruhe, Germany). The filtrate was finally dried under a fume hood until the solvent was completely evaporated. The recrystallization of wax structures on clean glass (cover glass, 18 × 18 mm) was carried out by PVD, resulting in a wax coating of the complete glass. The device for recrystallization (Figure 2) consisted of a glass chamber (Vakuum- und Industrieservice Meier GmbH, Borken, Germany), a holder for the samples to be coated, a flat heater made of high-performance ceramics (40 Ω, BACH Resistor Ceramics GmbH, Werneuchen, Germany), a laboratory power supply unit, and a vacuum pump (Duo 5M, Pfeiffer Vacuum GmbH, Asslar, Germany). To create a vacuum of 4 × 10^−3^ mbar, air was pumped out for 30 min. The amount of wax deposited on the samples can be varied by the distance between the evaporation point and the sample or by changing the amount of wax to be evaporated. Here, three different amounts of wax (small amount: 700 µg, medium amount: 1400 µg, and large amount: 2800 µg) were placed on the heating plate heated up to 200 °C and evaporated to obtain different wettabilities of the samples. The distance between the heating plate with the wax source and the samples to be coated was 4 cm. After wax deposition, samples were stored in an oven at 50 °C for 72 h. In the following, the artificial surfaces are named according to the amount of wax used for wax coating.

### Scanning electron microscopy of wax morphology

The morphologies of the wax on the upper (adaxial) and lower (abaxial) leaf sides (leaf 2, 3, and 4, *n* = 3) as well as of recrystallized wax structures on glass (*n* = 3) were analyzed by scanning electron microscopy (SEM, Gemini Supra 40 VP, Zeiss, Oberkochen, Germany). The middle part of fresh wheat leaves was cut into small pieces (approx. 0.3 × 0.5 cm) using a scalpel and attached to aluminium SEM sample holders (diameter 2.4 cm, Plano, Wetzlar, Germany) with conductive double-sided adhesive tape (Leit-Tabs, Plano, Wetzlar, Germany). The samples were coated with a thin gold layer (99.9% purity, approx. 8 nm) using a sputter coater (Cressington 108 auto SE, Elektronen-Optik-Service GmbH, Dortmund, Germany; 60 s, 30 mA, 0.1 mbar)*.* Glasses coated with wax were mounted and sputter-coated in the same way as the wheat leaf pieces. SEM analysis of all samples was carried out at a voltage of 10 kV using the secondary electron detector. The working distance was 4–7 mm.

### Atomic force microscopy analysis of recrystallized structures

The thickness of the wax coating on glass (1400 µg) was examined with an atomic force microscope (AFM, NanoWizard II, JPK instruments, Berlin, Germany). For this purpose, the recrystallized wax layer on the glass was partially removed with a razor blade. The sample was then attached to a microscope slide with double-sided adhesive tape. The AFM recordings were performed in tapping mode (amplitude: 0.05 V; frequency: 302.9 kHz, line rate: 0.3 Hz, set point: 940 mV) with tapping cantilevers (Tap300-G, Budget Sensors, Sofia, Bulgaria). The acquired height and amplitude images (50 × 50 µm) were processed and analyzed using JPK´s data analysis software (JPK Data Processing, version 4.2.62). At the edge of the wax layer, the height differences between the wax layer and the uncoated glass were measured using the cross-section tool (*n* = 20).

### Analysis of the chemical composition of wax

Fresh leaves were cut from intact plants and immediately processed. To extract the epicuticular waxes of the greenhouse plants, three leaves per leaf age were combined for one replicate and dipped for 20 s in 20 mL chloroform. Five independent biological replicates were analyzed (*n* = 5). For the extraction of epicuticular wax from outdoor plants, leaf 2, 3, and 4 were pooled together for one replicate. Here the analysis of six independent biological replicates was performed (*n* = 6). Leaves were scanned immediately after wax extraction using a scanner (LA2400 with Winrhizo, Regent Instruments Inc., Québec, Canada) to determine the leaf areas. Tetracosane (C_24_ alkane, *c* = 0.2 mg·mL^−1^, Merck KGaA, Darmstadt, Germany) was added to 50 µL of each extract as an internal standard. An aliquot of each wax extract was taken for chemical analysis. The volumes of the aliquots were reduced to 200 µL at 70 °C under a stream of gaseous nitrogen. To ensure that the wax composition did not change due to the evaporation process, the chemical composition of the wax coating of the artificial surfaces (1400 µg) was analyzed too (*n* = 4). For this purpose, the wax was stripped with collodion (4%, Merck KGaA, Darmstadt, Germany). The collodion strip was then placed in 10 mL chloroform and 100 µL tetracosane was added as an internal standard. All samples were derivatized with 20 µL bis(trimethylsilyl)trifluoroacetamide (BSTFA) for 45 min at 70 °C. 20 µL of pyrimidine (Merck KGaA, Darmstadt, Germany) was used as catalyst. The waxes were analyzed quantitatively with a gas chromatograph coupled with a flame ionization detector (GC–FID, 6890N, Agilent Technologies Sales & Services, column: DB-1; 30 m × 0.32 mm, 0.1 μm; J&W, Agilent Technologies Sales & Services GmbH & Co. KG, Waldbronn, Germany). The qualitative analysis of the wax components was carried out with a gas chromatograph coupled with a mass spectrometer (GC–MS, 5973, Agilent Technologies Sales & Services GmbH & Co. KG, Waldbronn, Germany). The individual wax monomers were identified by their fragments formed by electron impact ionisation using HP-Chemstation software (Hewlett Packard Cooperation, Palo Alto, USA). The injection volume was 1 µL and the carrier gas was hydrogen for GC–FID and helium for GC–MS.

### Analysis of the wetting properties

The wetting properties of wheat leaves (OD) and glass with and without wax coating were analyzed by determining the static CA, the TA, and the CAH of a water droplet (aqua dest., droplet volume: 5 µL; *n* = 15). For this purpose, a goniometer (OCA 35, DataPhysics Instruments GmbH, Filderstadt, Germany) equipped with a digital camera (RM-6740CL, JAI, Copenhagen, Denmark) was used. The CAH was determined on an inclined plane just before the droplet rolled off (tilting plate method). The CAs were measured in the first frame of the videos in which the CAH and the TAs were determined. The leaves were tilted in a way that the drops rolled off longitudinally to the leaf venation. If a drop rolled off immediately after deposition, the CA and the CAH were not measurable. The TA was set to 0.1° in these cases. The wetting properties of leaf 2, 3 and 4 and of their upper and lower sides were analyzed separately (for each leaf *n* = 15).

### Utilization of the artificial leaf surface

To comparatively investigate the biotic interactions of the artificial leaf surfaces, wheat leaves and the artificial leaf surfaces (1400 µg) were inoculated with conidia (asexual spores) of powdery mildew (*Blumeria graminis* (DC.) Speer f. sp. *tritici*, Erysiphaceae), a natural pathogen of wheat. Conidia from infested wheat plants (provided by Prof. Ulrich Schaffrath, Lehrstuhl und Institut für Biologie III (Pflanzenphysiologie), RWTH Aachen, Germany) were cultivated on wheat plants (20 °C, 12 h/12 h day/night rhythm, 76% rel. air humidity). Infested plants used for inoculation were always shaken manually one day before inoculation to remove old conidia. This procedure should ensure that only fresh conidia are transferred during inoculation. For inoculation, freshly cut turgescent leaves (*n* = 5) and the artificial leaf surface (*n* = 10) were placed on the bottom of an inoculation tower (height: 80 cm, length: 25 cm, width: 25 cm). Plants whose leaves clearly showed powdery mildew pustules were then shaken out over the surfaces. After 1 h, the inoculated surfaces were removed from the inoculation tower and incubated in the dark at 20 °C on a wet paper towel in a petri dish to achieve 100% relative air humidity. The germination success of 100 conidia on the different surfaces was examined using a digital microscope (VHX-600 DSO, Keyence, Osaka, Japan). Leaf pieces were cut and decolorized with ethanol/acetic acid (3:1, v/v) for 24 h until the leaves were bleached. For this purpose, the leaf pieces were placed on papers soaked with the ethanol/acetic acid mixture. To fix the structures, the leaf pieces were then transferred to papers soaked with lactoglycerol (lactic acid/glycerol/H_2_O, 1:1:1.3, v/v/v; lactic acid: ≥95% ʟ-(+)-lactic acid, Carl Roth GmbH und Co. KG, Karlsruhe, Germany; glycerol: ≥99%, Carl Roth GmbH und Co. KG, Karlsruhe, Germany) for about 3 h. The fungal structures were then stained with trypan blue (Sigma-Aldrich, St. Louis, USA, 0.05% in lactoglycerol).

### Data analysis

The data were statistically analyzed with the help of the software R Studio (R Core Team (2021). R: A language and environment for statistical computing. R Foundation for Statistical Computing, Vienna, Austria. URL https://www.R-project.org/). To evaluate significant differences (*p* < 0.05) in the wax composition and in the wetting properties between natural and artificial surfaces, a Kruskal–Wallis test followed by a post hoc Dunn test was performed. Differences in the wax composition between leaves 2, 3, and 4 were determined by an ANOVA test followed by a post hoc Tukey test.

## Results

### SEM analysis of wax morphology

**Figure 3:**
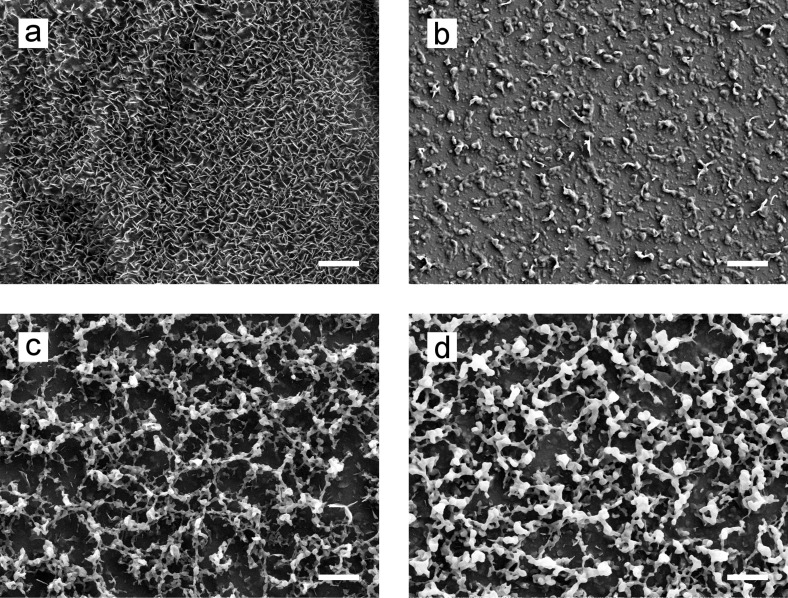
SEM micrographs of wax structures. (a) Natural leaf surface of wheat, (b–d) artificial surfaces coated with different evaporated wax masses: 700 µg (b); 1400 µg (c); 2800 µg (d); scale bars: 4 µm. On the natural surface, wax platelets in a dense arrangement were visible. On all artificial surfaces, granule-shaped structures were observed.

SEM analysis showed that wheat leaves were covered with wax platelets (Figure 3a). The platelets had irregular edges and were very close to each other. Both leaf sides, leaves of different ages, and leaves from greenhouse and outdoor plants showed the same wax structures (Supporting Information File 1, Figure S2). The surface of the plants grown outdoors showed alterations in the wax layer (Supporting Information File 1, Figure S3).

**Figure 4:**
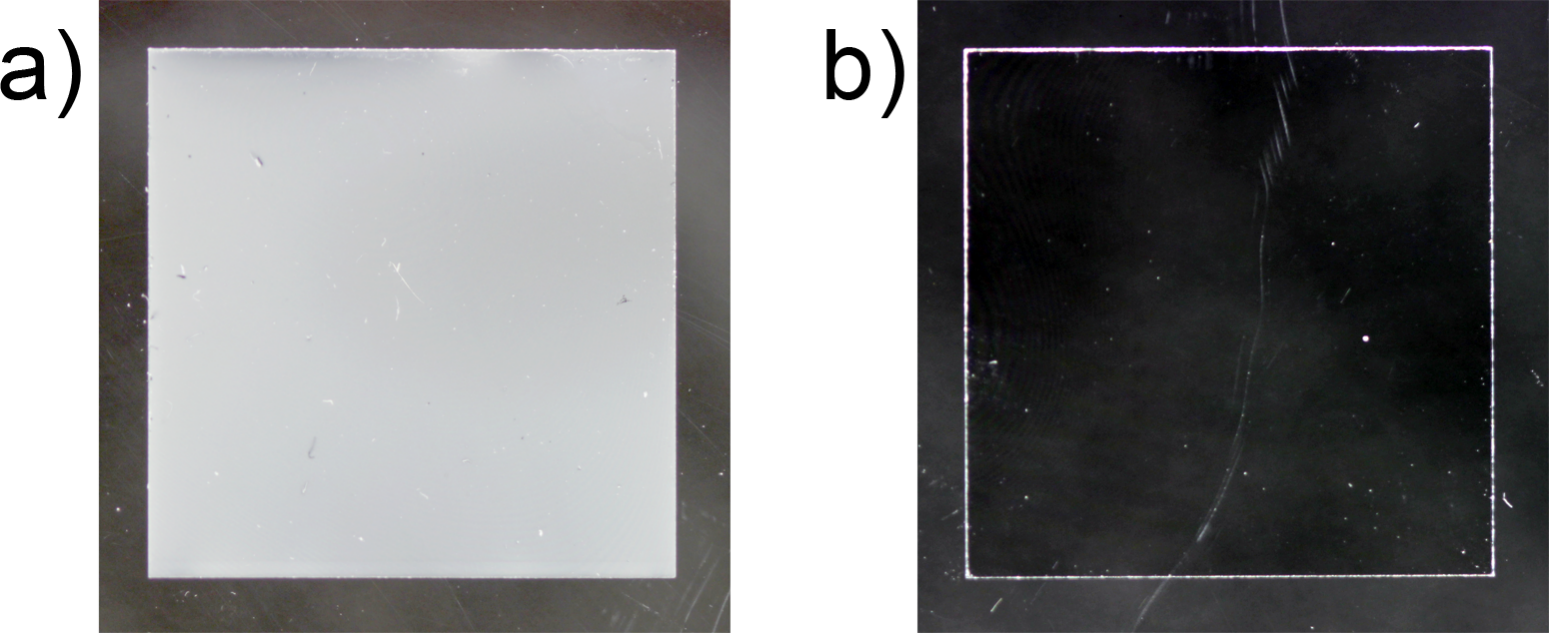
Photographs of glass cover slides before and after coating. (a) Pure non-treated glass (18 × 18 mm), (b) glass coated with wheat wax (1400 µg).

Macroscopically, vapor deposition with the wheat wax resulted in a white opaque homogeneous coating on the glass (Figure 4). SEM images showed three-dimensional, granularly recrystallized structures on all wax-coated artificial surfaces, with a homogenous distribution (Figure 3b–d). With increasing evaporated wax mass, the amount of wax and especially the layer thickness of the recrystallized structures increased. The structures were interconnected and formed a net-like structure with infrequent scales between them.

### AFM analysis of recrystallized structures

**Figure 5:**
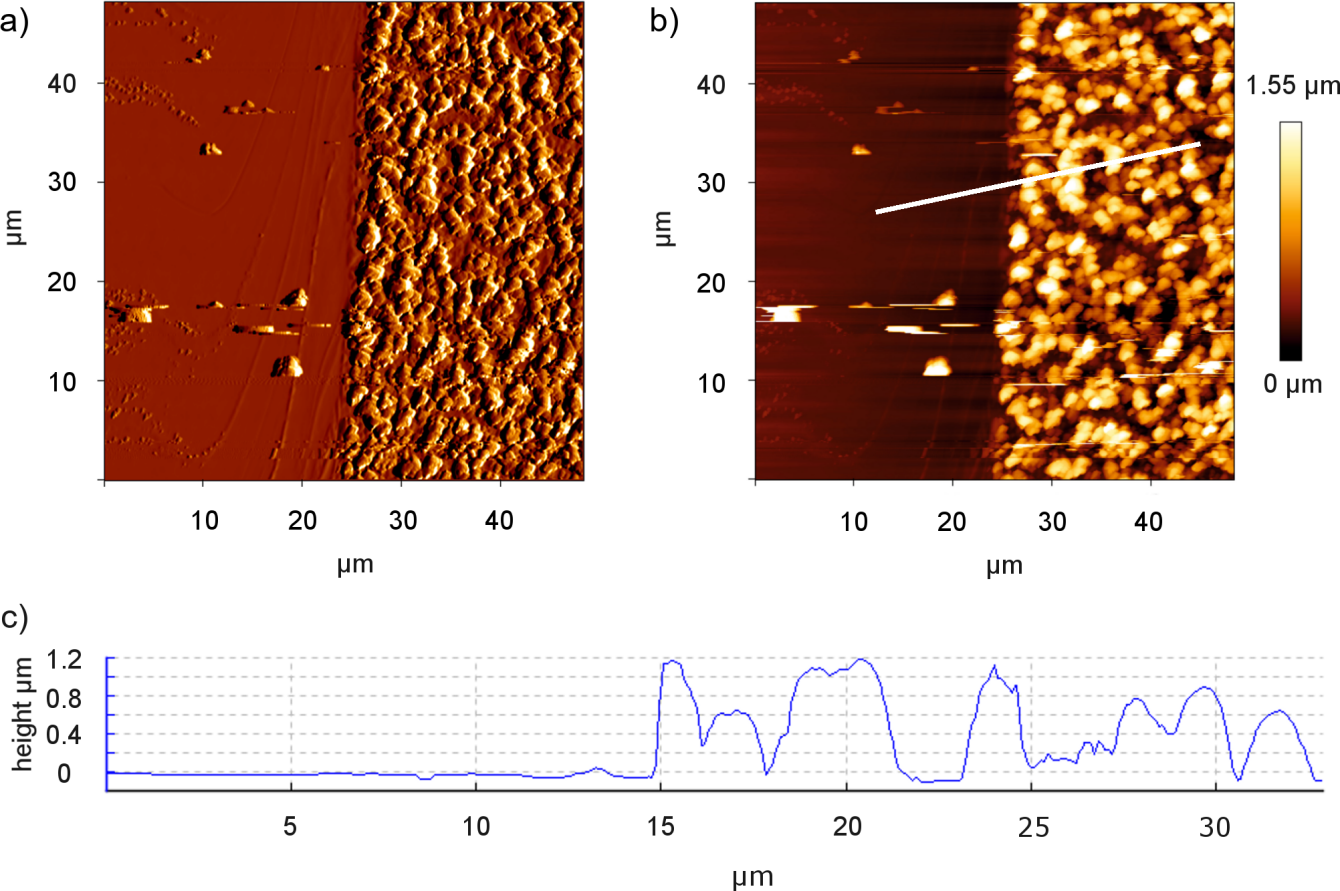
Results of AFM investigation of the wheat wax layer on glass (artificial leaf surface; 1400 µg). (a) Amplitude image, (b) height image. The white line indicates where the cross section in (c) was made. (c) Cross section. On the left side of each image: glass after the removal of the coating, on the right side: structure of the wax coating.

Like the SEM analysis, the AFM analysis showed granule-shaped structures on the artificial surface (1400 µg). By removing the wax with a razor blade, a distinct edge was created, showing a 1.12 ± 0.23 µm thick wax layer (Figure 5).

### Analysis of chemical composition of wax

**Figure 6:**
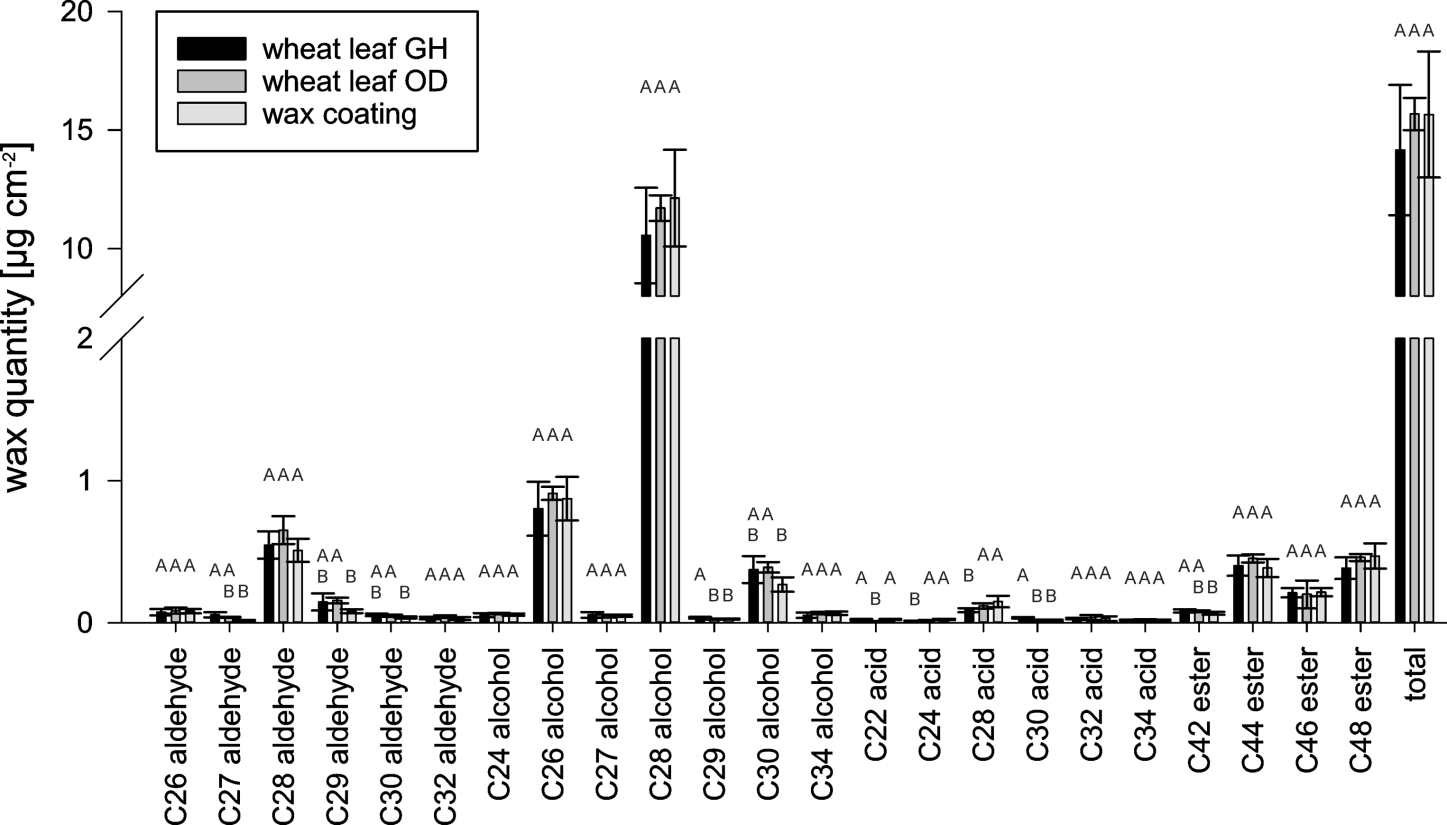
Chemical composition of wheat wax. GH: greenhouse plants (*n* = 15); OD: outdoor plants (*n* = 6). Wax coating: wax layer of a wheat wax-coated glass (*n* = 4). Different letters indicate significant differences in the amounts of the wax components between wheat leaves from GH, OD, and the artificial leaf surface (*p* < 0.05, tested by a Kruskal–Wallis test followed by a post hoc Dunn test).

The wax monomers and the amount of wax per square centimeter leaf area of wheat leaves were chemically analyzed. The amount of wax increased with the decreasing age of the leaf (leaf 2: 11.5 ± 1.2 µg·cm^−2^, leaf 3: 13.6 ± 0.8 µg·cm^−2^, and leaf 4: 17.3 ± 1.5 µg·cm^−2^). The wax compositions of leaves 2, 3, and 4 of the greenhouse plants were analyzed separately to investigate age-specific differences, but only slight differences were detected in the relative wax composition (Supporting Information File 1, Figure S4). Therefore, the wax of leaves 2, 3, and 4 was not further separated for the subsequent recrystallization experiments. The total amounts of wax of green house plants and outdoor plants were the same with 14.2 ± 2.8 and 15.7 ± 0.7 µg·cm^−2^, respectively (Figure 6). The comparison of the wax composition of greenhouse plants and outdoor plants showed no significant differences in the amounts of the substance classes. Both waxes were mainly composed of alcohols (GH: 11.9 ± 2.4 µg·cm^−2^, OD: 13.2 ± 0.6 µg·cm^−2^). The main component was 1-octacosanol (GH: 10.6 ± 2.0 µg·cm^−2^, OD: 11.7 ± 0.5 µg·cm^−2^). Besides the alcohols, small amounts of esters (GH: 1.0 ± 0.2 µg·cm^−2^, OD: 1.2 ± 0.1 µg·cm^−2^) and aldehydes (GH: 0.9 ± 0.2 µg·cm^−2^, OD: 1.0 ± 0.14 µg·cm^−2^) were detected. Acids were detected in traces (GH: 0.2 ± 0.02 µg·cm^−2^, OD: 0.2 ± 0.04 µg·cm^−2^).

The chemical composition of the wax coating was checked to determine whether the wax composition had changed during the coating process. The wax coating of an artificial leaf surface was composed of the same substances in comparable amounts as the waxes of the natural leaves. The total amount of wax on the coated artificial surface (1400 µg) was, with a value of 15.6 ± 2.6 µg·cm^−2^, the same as for the wheat leaves. The amount of alcohols (13.5 ± 2.2 µg·cm^−2^), esters (1.1 ± 0.2 µg·cm^−2^), aldehydes (0.8 ± 0.1 µg·cm^−2^), and acids (0.3 ± 0.07 µg·cm^−2^) did not differ significantly from the amounts found in wax of wheat leaves. 1-octacosanol was, with a value of 12.1 ± 2.0 µg·cm^−2^, also the main component. Significant differences in the amounts of single wax components between the waxes of greenhouse plants, of outdoor plants, and wax-coated artificial leaf surfaces were only found for substances with amounts below 0.5 µg·cm^−2^.

### Analysis of wetting properties

**Figure 7:**
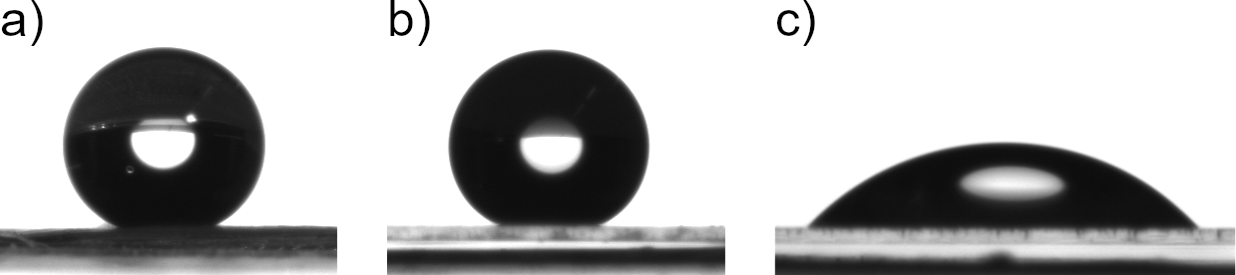
Goniometer photographs of water droplets. (a) On a turgescent wheat leaf (CA = 144°), (b) on a wax-coated artificial surface (1400 µg, CA = 155°), and (c) on clean non-treated glass (CA = 46°); all mentioned CAs are the individual CAs of the water droplets shown in the images.

All wheat leaves were highly hydrophobic (leaf 2, 3, and 4) on the upper and the lower leaf sides. The deposited water droplets appeared spherical (Figure 7a). The CAs of water ranged between 127.5° ± 13.6° and 147.5° ± 11.7°. The deposited droplets rolled off from all leaves at TAs ranging from 10.8° ± 7.3° up to 29.4° ± 15.6°. The CAH values varied from 8.2° ± 8.3° to 25° ± 10.3° (Table 1). The CA, TA, and CAH values of the upper surface of leaf 3 were compared with the values of the wax-coated artificial surfaces for the following experiments.

**Table 1:** Wetting properties of wheat leaves (OD, mean ± SD, *n* = 15).^a^

Sample	CA [°]	TA [°]	CAH [°]

leaf 2 upper side	143.6 ± 9.3	26.2 ± 14.1	22 ± 10.6
leaf 3 upper side	147.5 ± 11.7	15.7 ± 10.8	8.2 ± 8.3
leaf 4 upper side	141.7 ± 11.8	10.8 ± 7.3	9.0 ± 8.3
leaf 2 lower side	139.8 ± 17.5	28.6 ± 14	25.0 ± 10.3
leaf 3 lower side	140.5 ± 15.6	29.4 ± 15.6	23.1 ± 12.5
leaf 4 lower side	127.5 ± 13.6	31.3 ± 21.3	23.2 ± 12.7

^a^CA: contact angle, TA: tilting angle, CAH: contact angle hysteresis.

**Figure 8:**
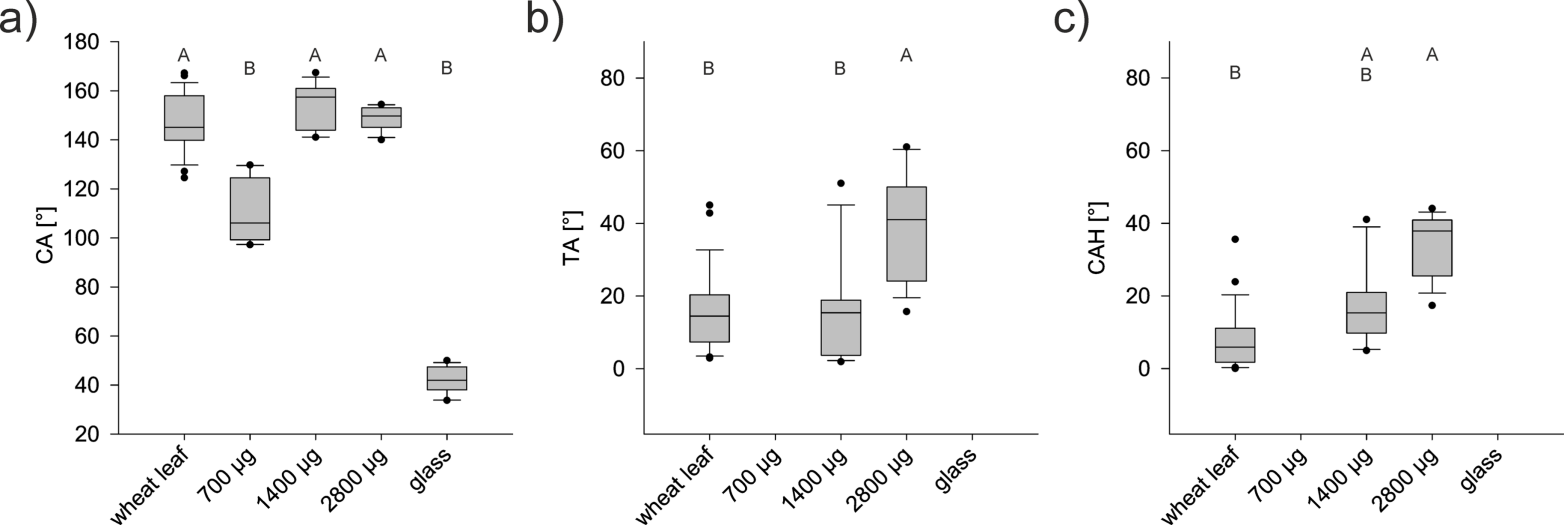
Wetting properties of the natural wheat leaf and the artificial surfaces covered with 700, 1400, or 2800 μg recrystallized epicuticular wheat wax. (a) Contact angle (CA), (b) tilting angle (TA), and (c) contact angle hysteresis (CAH) of water droplets (5 µL). The droplets on glass did not roll off, hence, neither TA nor CAH were measurable. Different letters indicate significant differences between the values of the different surfaces (*p* < 0.05, tested by a Kruskal–Wallis test followed by a post Hoc Dunn test).

The artificial surfaces were also hydrophobic (Figure 7b). In contrast to clean, non-treated glass (Figure 7c), the CA of the wax-coated surfaces were all in the hydrophobic range. The smallest CA, 110.1° ± 12.0°, was measured on the artificial surface, on which the small wax amount was evaporated. The CAs of the artificial samples with medium (154.7° ± 8.8°) and with large amounts of evaporated and recrystallized wax (148° ± 4.7°) did not differ from those of the wheat leaves (147.5° ± 11.7°) (Figure 8a).

As on clean, non-treated glass, the droplets did not roll off the artificial surfaces with small amounts of wax. Therefore, neither TA nor CAH could be measured on these surfaces. On the artificial surfaces with the large amount of wax, the droplets only rolled off at higher TA (38.9° ± 14.6°) than on the wheat leaves (15.7° ± 15.1°). The TA of the artificial surfaces covered with the medium wax mass (14.9° ± 13.8°) was the same as on the wheat leaves (Figure 8b). The CAH of artificial surfaces with the medium wax mass (17.6° ± 10.6°) was also the same as that of the wheat leaves (8.2° ± 8.3°). The CAH of the artificial surfaces covered with the high wax mass was higher (33.5° ± 8.8°) than that of the natural surfaces (Figure 8c).

### Utilization of the artificial leaf surface

**Figure 9:**
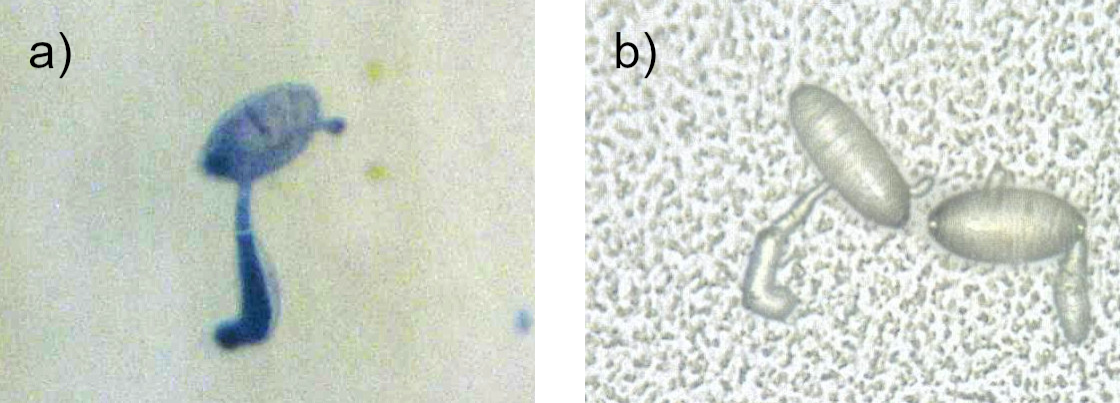
Digital microscopy images of powdery mildew conidia at 1000× magnification. (a) On a wheat leaf, stained with trypan blue, (b) on the artificial leaf surface. Fungal conidia differentiated on both surfaces, clearly indicated by the development of the hooked appressorium.

Since the artificial surface with the medium wax mass had the same wetting properties as the wheat leaves, this artificial surface was selected as “artificial leaf surface” for an inoculation experiment with fungal spores. For this purpose, wheat leaves and the artificial surfaces were inoculated with conidia of wheat powdery mildew. The microscopic images show that the fungal spores germinated and differentiated on both the natural and the artificial surface (Figure 9). The germination rates were 76.8% ± 13.2% on the wheat leaf and 48.6% ± 12.6% on the artificial leaf surface.

## Discussion

Plant surfaces have specific physicochemical properties due to the cuticle and, in particular, due to the epicuticular waxes deposited on it. In previous studies, bio-inspired technical surfaces were often used with surface properties only partially corresponding to those of the natural surfaces. In the present work, an artificial leaf surface was developed that corresponded to the plant leaf surface in its chemical properties and its wettability.

### Wax morphology

#### Natural surface

First, the surface properties of the natural wheat leaf were characterized. For wheat leaves, platelets as well as tubules have been previously described as wax structures [17,20,49–52]. Further, the wax structures can vary depending on the developmental stage of the plant [49]. SEM investigation showed platelets on all leaves of different ages (Figure 3a, Supporting Information File 1, Figure S2). This result meets the expectations, as tubules were described for other plant organs such as leaf sheets, stems, and glumes [50–51,53–54], or only on the abaxial sides of the glaucous flag leaves of plants at a later stage of development [50–51]. In our study, the structures of both leaf sides were the same (Supporting Information File 1, Figure S2). This is in accordance with other studies. In Koch et al. [20] and Wang et al. [53] plants in a comparable developmental stage were investigated. A network of densely packed wax platelets was also described for both sides of the leaf [53].

Abiotic factors such as temperature, humidity, pollution, and radiant energy might cause changes in the wax morphology [55–57]. To take this into account, wheat plants were also cultivated outdoors under natural growth conditions. No culture-specific differences in the wax morphology were observed. As with the greenhouse plants, the leaves of the plants cultivated outdoors were covered with wax platelets. Hence, the wax morphology of the investigated wheat leaves showed no developmental and ecological variability. The wax platelets could therefore be used as a native model for the development of artificial leaf surfaces.

#### Artificial surfaces

As a decisive prerequisite for the applicability of the artificial surface as bio-inspired leaf surface, it was defined that the artificial surface needs to have a homogeneous coating and uniform wettability. Previous recrystallization studies have shown that a homogeneous coating can be produced by thermal evaporation [40,46,58–60]. In the present work, a homogeneous coating was also achieved with this method, as can be seen in the photograph (Figure 4) and in the SEM micrograph (Figure 3). The latter showed that the wax recrystallized to granular structures with single plates between the granular network. However, after deposition of the lowest wax mass, almost no plates were formed, indicating that the polar and amorphous substrate prevents the formation of a platelet structure. In contrast, higher wax masses lead to a growth of the platelets on the wax deposited below. It was clearly visible that the individual plates were not formed directly on the substrate, but stood on a granular layer. Koch et al. [20] demonstrated that on non-polar substrates, such as highly ordered pyrolytic graphite, wax composed of primary alcohols recrystallize into platelets, as on the wheat leaves. Even though the recrystallized structures in the present study did not exactly match the native wax structures, the wetting properties of the artificial leaf surfaces were similar to those of the natural leaf. The glass substrate used here offers an easy-to-handle and inexpensive way to develop large numbers of wax-coated samples with wetting properties that are similar to that of the natural leaf.

### Wax chemistry

#### Natural surface

Next to the wax morphology, the wax chemistry of wheat leaves was analyzed, as it plays an important role in the functionality of the leaf surface. The extracted wheat wax was composed of alcohols, aldehydes, esters, and acids. The main component was 1-octacosanol (Figure 6). The results are in agreement with previous studies on the wax chemistry of other wheat varieties [20,50,61–62]. β-Diketone, which is a component of wax tubules, has so far only been detected in wax of wheat leaves at later developmental stages and other plant organs [50,53,61,63–66]. Alkanes, which were detected in wheat wax in some other studies, were not a component of the wax examined here [20,52–53,62–64].

Developmental changes can have an influence on the wax composition [67–68]. Therefore, the composition of epicuticular waxes of leaves of different ages was analyzed in the greenhouse-cultured plants to examine whether there were any differences. The total wax amount decreased from leaf 2 to leaf 4, that is, from older to younger leaves. The decrease in wax amounts was evenly distributed over all substance classes, so that there were no notable differences in the relative wax composition and the corresponding wax morphology (Supporting Information File 1, Figure S4).

Abiotic factors can have an influence on the amount of epicuticular wax. For example, previous studies have shown that higher temperatures, reduced humidity, increased UV radiation, or drought stress can lead to increased wax accumulation [55,64,69–71]. Therefore, it was also examined whether there were differences in the wax chemistry between the greenhouse plants and the outdoor plants. The results showed hardly any differences between the differently cultivated plants. The total wax amount and the amounts of the single substance classes were the same (Figure 6). Consistent with the results of the wax morphology study, the wax composition of wheat appears to be strongly genetically fixed and little influenced by environmental factors. Thus, the wax extracts of plants growing outdoors or indoors can be used for the development of artificial leaf surfaces.

#### Artificial surfaces

To make sure that wax composition had not changed during the coating process, the wax layer of a coated artificial leaf surface was analyzed. By comparison of the native wax with the wax recrystallized after thermal evaporation, we could show that the wax still had the same chemical composition as the wax on the native leaves. In the wax precipitate on glass, the same substances were detected in the same amounts as in the wax that was vaporized (Figure 6). Thus, no transformation of substances had taken place and the wax composition was stable. In a previous study, the wax composition of leek wax was also found to be thermally stable [72].

Since the evaporated substances spread uniformly in vacuum from the point source and the samples were placed in an arc above the substance source, in previous studies, the wax coverage was calculated approximately from the quotient of the evaporated masses and the surface area of a hemisphere [42,58,72–73]. The analysis of the wax coating of the evaporated surface performed here, in contrast, allowed for an accurate measurement of the wax coverage. At 15.7 ± 2.6 µg·cm^−2^, it was the same as the wax coating of a wheat leaf (Figure 6). The wax coating of the technical surface thus corresponded in its chemical properties both qualitatively and quantitatively to the wax coating of the natural surface.

### Wetting properties

#### Natural surface

The wetting behavior of a plant surface is relevant for the interaction of the plant with its biological and non-biological environment. For instance, surface moisture plays a crucial role in the development of fungal diseases in plants [74]. Also, the wettability has to be considered in the context of the application of agrochemicals [75]. Thus, as a criterion for success it can be postulated that an artificial in vitro test system should reflect the wettability of the native surface. Consequently, in addition to wax morphology and wax chemistry, the wettability of wheat leaves was investigated.

Often, only the CAs are used to describe the wettability. However, specifying CAs alone to describe wettability can be misleading and insufficient [75–76]. For example, a droplet with a high CA may stick to an inclined surface or roll off easily, depending on the wettability state. Therefore, the CAH and the TA of wheat leaves were also determined the present work.

The investigated wheat leaves were all hydrophobic (Table 1), which is in agreement with results of previous studies [29,50,77–78]. The CAH values ranged from 8.2° ± 8.3° to 25.0° ± 10.3° and the TAs ranged from 10.8° ± 7.3° to 31.3° ± 21.3° (Table 1). Wheat leaves are not vertically oriented to the plant axis. TAs provide information on droplet adhesion on such inclined surfaces. For wheat leaves, we could not find any comparative values for TAs in the literature. In the context of active ingredient application and uptake, Peirce et al. [78] investigated the retention of applied water droplets on wheat leaves. In this study, high CAs and very low CAH were also found, which indicates the ability for self-cleaning. However, the investigated plants were cultivated in the greenhouse. Weather-related damage to the leaves of the outdoor plants examined in the present study possibly caused the larger CAH and TAs and the high standard deviations.

Droplets generally rolled off rapidly from the wheat leaves. Microscopic images taken during the wetting measurements indicated that the water droplets did not completely fill the space between the anticlinal cell walls. Both observations suggest Cassie wetting or intermediate wetting. Cassie wetting was also assumed in a previous wettability study on wheat leaves [78]. In individual specimens, however, the water droplets remained attached to the surface and did not roll off even at a TA of 90°. In such samples, the wax layer was probably damaged or contaminated. Both wax alteration and contamination can lead to a change in wettability [75,79]. No damage or contamination was visible to the naked eye in the leaves used. SEM images of the leaves from the field cultures, however, showed clear damage in the wax layer (removed and flattened crystals) on the leaf surfaces in some cases (Supporting Information File 1, Figure S3). Presumably, a transition to Wenzel wetting takes place at damaged or dirty areas, so that the droplets no longer roll off. In the case of a drop on a rough surface, liquid is pressed between the cavities by the Laplace pressure. If the liquid penetrates into the cavities so far that it touches the substrate, a transition of the wetting states takes place [75]. Whether a transition occurs or not is influenced by the shape and dimensions of the supporting structures and by the droplet size [80]. In the present study, the droplet volume was always the same, but the structures determining the wetting may have been destroyed or masked by particles such that a transition could take place. To evaluate the wetting properties, it is advisable to use plants from greenhouse cultures, as their surfaces are less heterogeneous. The experiments in this work have shown that wax morphology and wax composition were barely influenced by the cultivation conditions (Figure 6). Nevertheless, plants in greenhouse cultures are better protected from phytopathogens and from wind and rain abrasion of wax. In this work, however, the wettability of outdoor grown plants was studied to be close to natural conditions.

#### Artificial surfaces

In this work, an artificial leaf surface was developed which, in addition to the same wax chemistry, also should have the same wetting properties as a natural leaf. This goal was achieved by vapor deposition with the medium amount of the wax extract. The surface coated in this way resembled the natural surface regarding all three studied wetting parameters (Figure 8).

The chemical analysis had shown that the wax composition of the wax coating on glass corresponds to that of the natural leaf surface. However, not only the wax chemistry but also the wax structure plays a role for the wettability. The different amounts of wax used made it possible to vary the structures and, thus, the wettability of the artificial surfaces. For example, the CA of the artificial surface covered with the small amount of evaporated wax was smaller than that of the wheat leaf. Hence, these artificial surfaces were more wettable than the natural ones (Figure 8a). Water droplets deposited did not roll off even at TAs of up to 90°. Presumably the water drops are not on the structures but sink in between them, resulting in Wenzel wetting [33]. On microstructured Si wafers, it could be shown that, depending on the height and the distance of the supporting columns of the surface, a transition from Cassie wetting to Wenzel wetting takes place. These and other criteria for the transition of a wetting stage were summarized by Bhushan and Jung [73].

The artificial surfaces vapor-deposited with 1400 and 2800 µg wax were as hydrophobic as the wheat leaves. However, the wax coating of the surface coated with 2800 µg appeared to be unstable. It seemed that rolling droplets carried wax particles on their surface and left a slight trace in the coating (data not shown). No loss of wax was observed on the artificial surface that was vapor-deposited with 1400 µg wax.

### Utilization of the artificial leaf surface

Here, an artificial leaf surface was developed that mimics the physicochemical properties of a natural leaf surface. The morphology of the recrystallized structures was not a 1:1 copy of the native structures, but the properties were still transferred. This proves that the thermal evaporation process made it possible to transfer the surface properties of the native surface to the technical surface. To the best of the authors’ knowledge, no artificial surface exists to date that reflects the wetting properties in addition to the chemistry of the epicuticular waxes. In previous studies, sophisticated systems provided with wax mixtures or with individual wax components were used to investigate the germination behavior of fungal spores [62,81–83]. These had the same wax chemistry, but the wettability was higher than that of the leaf surface. Also in this study, fungal spores were applied to the artificial leaf surface as a first application test. The microscopic analysis showed that the spores germinate and differentiate on the artificial leaf surface. Even though the number of germinated spores was lower than on the natural leaf, the spores were able to germinate on our artificial leaf surface. Hence, the here developed artificial leaf surface offers an opportunity to study interactions of the plant surface ex situ. In contrast to the natural leaf, the artificial leaf surface is less complex and easily adjustable.

## Conclusion

In this work, an artificial leaf surface was developed by vapor depositing a wax mixture on glass substrates, which mimics the physicochemical properties of a natural leaf. The thermal evaporation process allowed for a homogeneous coating of the glass with three-dimensional structures. By coating with the medium amount of wax (1400 µg), the wettability and the wax composition of the wheat leaf surface could be successfully transferred to the technical surface. For native and artificial leaf surfaces, a detailed wettability and chemical analysis was performed to ensure that the artificial system represented the physicochemical characters of the natural leaf surface. With this biomimetic surface, a test system is available for the first time that represents both the chemical signals of the waxes and the wettability of the plant surface. Such an artificial leaf surface offers promising possibilities to investigate interfacial phenomena of the plant surface in detail under controlled conditions. In addition to study the germination of phytopathogenic fungi, other conceivable uses would be, for example, to study the application and retention of various liquids and active ingredients.

## Supporting Information

Supporting Information includes a photograph of a harvested wheat plant at the five-leaf stage (Figure S1) to show which leaves were used for the experiments, SEM micrographs of crystalline wax platelets on both sides of wheat leaves of different ages (Figure S2), a micrograph of damage in the wax layer of a wheat leaf of plant cultivated outdoors (Figure S3), and a diagram of the chemical composition of wheat wax of leaves of different ages (Figure S4).

File 1 Additional experimental data.
