# Supplementary material for: Design of a biomimetic, small-scale artificial leaf surface for the study of environmental interactions
Source: Beilstein J Nanotechnol. 2022 Sep 13;13:944–57. doi: 10.3762/bjnano.13.83 (PMC9490070; doi:10.3762/bjnano.13.83)
Supplement: File 1 — Additional experimental data. [file Beilstein_J_Nanotechnol-13-944-s001.pdf]

BEILSTEIN JOURNAL OF NANOTECHNOLOGY

Supporting Information

for

Design of a biomimetic, small-scale artificial leaf surface for the study of environmental interactions

Miriam Anna Huth, Axel Huth, Lukas Schreiber and Kerstin Koch

Beilstein J. Nanotechnol. **2022,** *13,* 944–957. doi:10.3762/bjnano.13.83

Additional experimental data

License and Terms: This is a supporting information file under the terms of the Creative Commons Attribution License (https://creativecommons.org/licenses/by/4.0). Please note that the reuse, redistribution and reproduction in particular requires that the author(s) and source are credited and that individual graphics may be subject to special legal provisions.
The license is subject to the *Beilstein Journal of Nanotechnology* terms and conditions: (https://www.beilstein-journals.org/bjnano/terms)

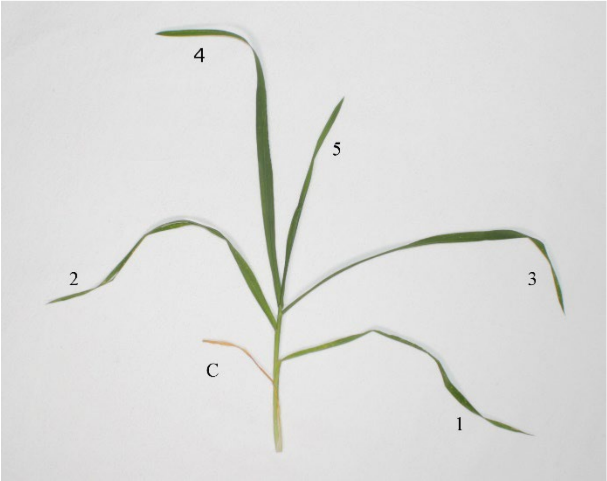

Figure S1: Harvested wheat plant at the five-leaf stage. C: cotyledon, 1–5: leaf 1–5; leaves used for the experiments: 2–4.

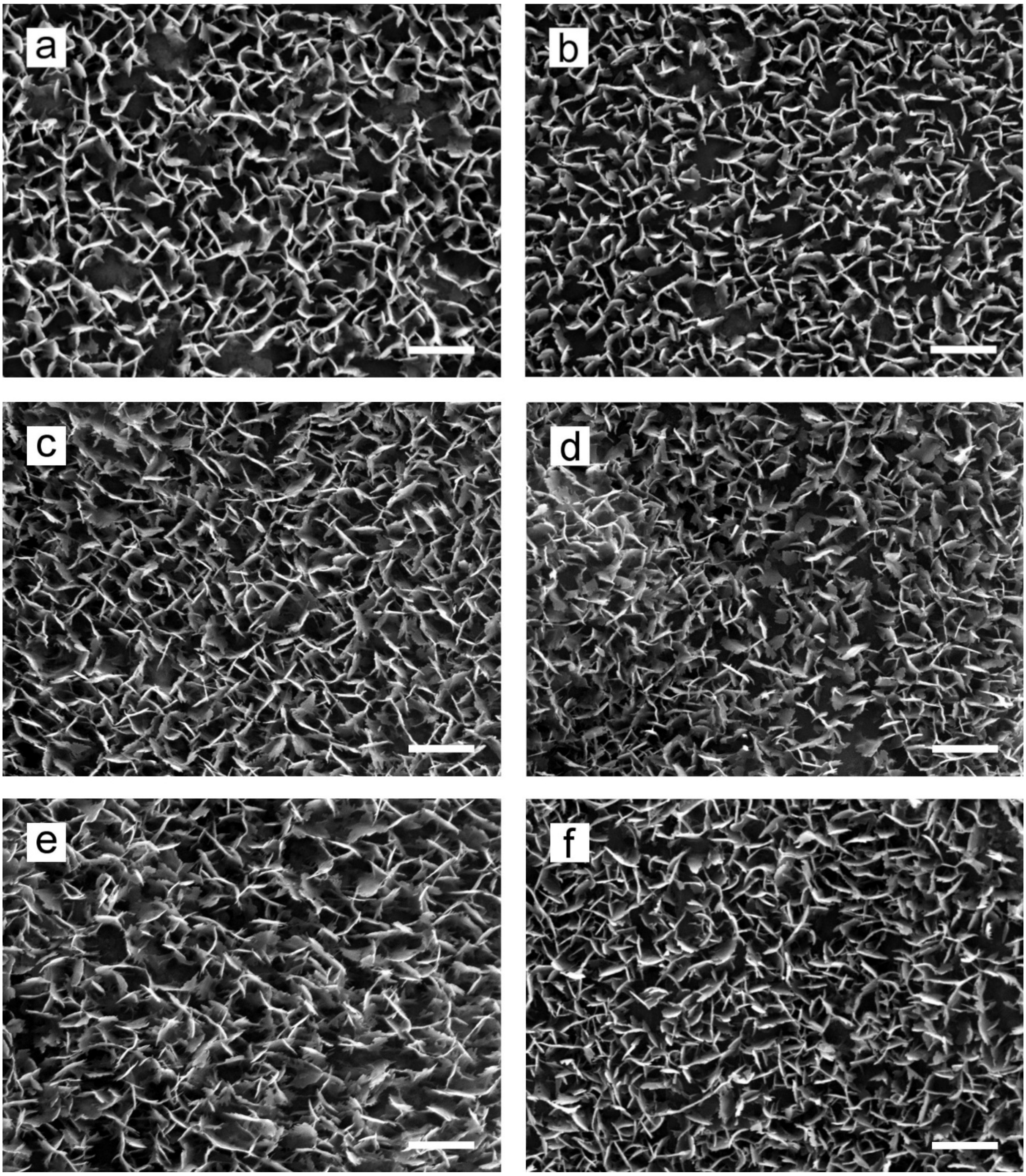

Figure S2: SEM micrographs of crystalline wax platelets on wheat leaves. (a) Upper (adaxial) side of leaf 2, (b) lower (abaxial) side of leaf 2, (c) upper side of leaf 3, (d) lower side of leaf 3, (e) upper side of leaf 4, (f) lower side of leaf 4; scale bars: 2 μm.

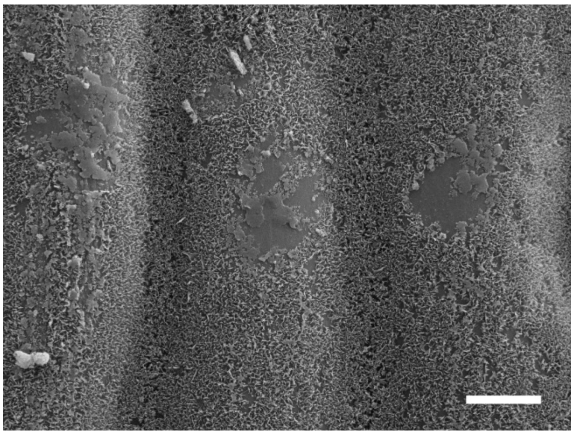

Figure S3: SEM micrograph of damage in the wax layer of a wheat leaf of an outdoor-cultivated plant. Scale bar: 10 µm.

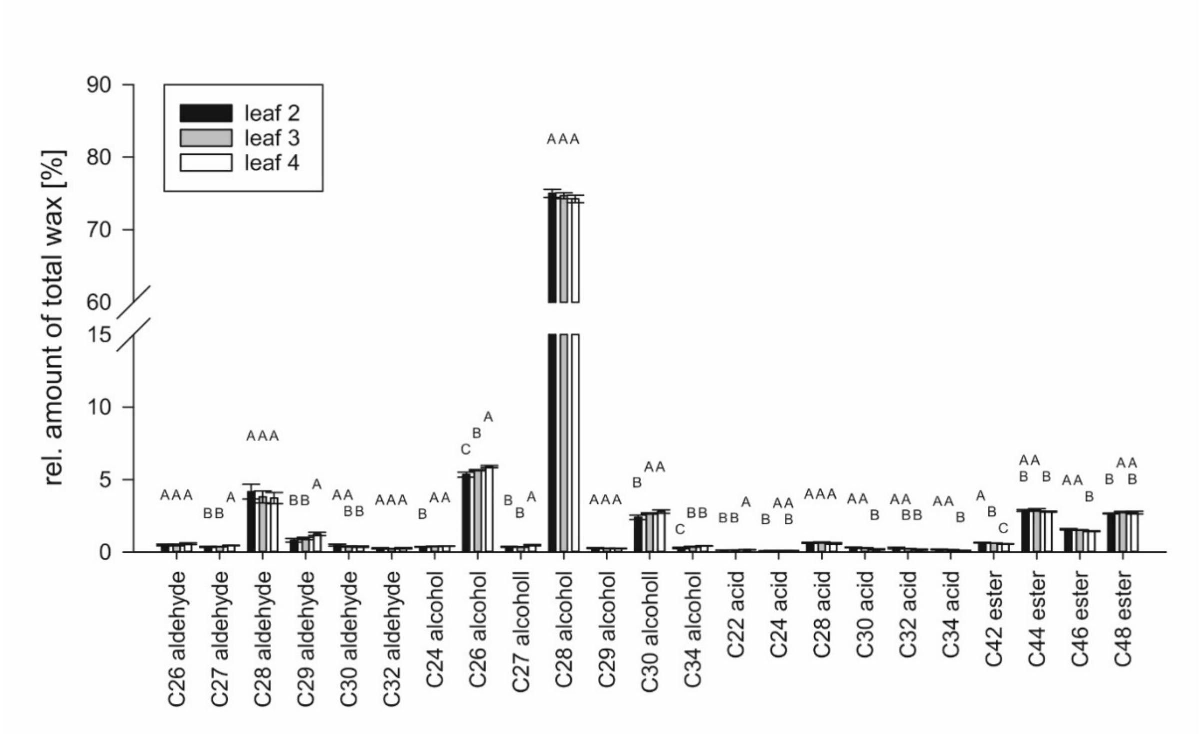

Figure S4: Chemical composition of wheat wax of leaves of different ages. Different letters indicate significant differences in the amounts of the wax components between leaves ($p < 0.05$, tested by an ANOVA test followed by a post hoc Tukey test).
